# Effects of *Mucuna pruriens* (L.) DC. and Levodopa in Improving Parkinson’s Disease in Rotenone Intoxicated Mice

**DOI:** 10.3390/cimb46080545

**Published:** 2024-08-22

**Authors:** Sheher Bano Zaigham, Dong-Guk Paeng

**Affiliations:** Department of Ocean System Engineering, Jeju National University, Jeju 63243, Republic of Korea; sheherbano7339@stu.jejunu.ac.kr

**Keywords:** Parkinson’s disease, *Mucuna pruriens*, rotenone, mitochondria, levodopa, IL-12, IL-6, TGF-*β*1

## Abstract

Parkinson’s disease (PD) is the second leading neurodegenerative disease after Alzheimer’s disease. *Mucuna pruriens* (L.) DC. (MP) is a plant that contains Levodopa (L-DOPA) and has been known to improve the symptoms of PD. In this preliminary study, we investigated the anti-parkinsonian potential of MP to compare the effects of L-DOPA. We first developed an in vivo model of the PD in C57BL/6 male mice using rotenone. A total of twelve mice were used for this experiment. Nine mice were injected with rotenone (28 mg/kg) daily for 28 days. The mice experiments were performed to validate the effectiveness of MP to treat PD. Synthetic L-DOPA in a ratio of 1:20 with MP was used as MP contains 5% L-DOPA by weight in it. MP and L-DOPA were injected for 19 days on a daily basis. Cognitive function was evaluated using beam balance and olfactory tests. Serum analysis was performed using serum enzyme-linked immunosorbent assay (ELISA) analysis test. IL-12, IL-6, and TGF-β 1 were evaluated to validate the PD inducement and treatment. The levels of IL-12, IL-6, and TGF-β1 (*p* < 0.0001) in the PD mice group were significantly higher than those in the control group. The PD mice also showed higher latencies in beam balance and olfactory tests (*p* < 0.0001) compared to the control group. Both MP and L-DOPA-treated groups showed alleviation in latencies in beam balance and olfactory tests and decreased neuroinflammation in ELISA analysis (*p* < 0.001). The results treated by MP and L-DOPA showed insignificant differences in their values (*p* > 0.05). This proved that the MP and L-DOPA had similar effects in improving the symptoms of PD when used in the ratio of 1:20. Furthermore, both MP and L-DOPA reduced the level of IL-6 and TGF-β1 in this study. It may be inferred that a reduction in the level of IL-6 and TGF-β1 eventually leads to a reduction in the Th17 cells. The pathogenic Th17 is thought to be present in virtually all chronic inflammatory disorders. This can be an interesting area of research in further understanding the immunological effect of MP in ameliorating PD symptoms.

## 1. Introduction

Parkinson’s disease (PD) is the second-most common neurodegenerative disorder, affecting 2–3% of the population of the world. It is a progressive neurological disorder characterized by motor and non-motor features [1,2]. It is caused by the degeneration of dopamine-producing neurons in the brain. Dopamine is a neurotransmitter that plays a crucial role in regulating the movement of the body [3]. PD can cause a range of motor symptoms, including tremors, stiffness, and difficulty with balance and coordination. Non-motor symptoms such as depression, anxiety, and sleep disturbances may also occur [4]. While the cause of PD is not fully understood, there is increasing evidence to suggest that neuroinflammation may play a significant role in the pathogenesis of the disease. Neuroinflammation involves the activation of the inflammatory processes in the brain, including the release of pro-inflammatory cytokines and the activation of microglia, the immune cells of the brain. This neuroinflammation contributes to the degeneration of the dopaminergic neurons in PD [5,6]. While inflammation is a fundamental immune response to protect neurons from neuronal damage, it can also exacerbate neuronal injury through its neurotoxic effect [7]. In PD, activation of microglia leads to the production of IL(Interleukin)-12, while oxidative stress and activation of astrocytes cause the release of cytokines such as IL-1β, IL-2, IL-4, IL-6, TGF-α, and TGF-β1 [8].

Much of the development and physiology of the central nervous system (CNS) is controlled by cytokines networks, which contribute to tissue inflammation if deregulated [9]. These cytokines play significant roles in coordinating the activities of innate and adaptive immune systems. In response to pathogen recognition, innate immune cells secrete cytokines. These cytokines send signals to the adaptive immune system about the nature of the pathogen and guide naïve T cells to differentiate into the appropriate T-cell subtypes required to clear the infection [10,11]. In this study, we focused on IL-6, TGF-β1, and IL-12 serum biomarkers to investigate their effects on the progression and treatment of PD. The motive behind choosing these specific biomarkers is their direct involvement with Parkinson’s disease and neuroinflammation, which is described in detail below. IL-6 is a pro-inflammatory cytokine that acts in the initiation of innate immune responses [12] and elevates in serum samples of patients with PD [13,14,15,16]. Another study described IL-6 as a state marker of PD [14,17]. It plays a critical role in the pathogenesis of inflammatory disorders and the physiological homeostasis of neural tissues [18]. IL-12 is another cytokine that plays an important role in the immune system by promoting the differentiation and proliferation of T cells and natural killer cells [19]. It is formed by the activation of microglia. Activated microglia in the substantia nigra and putamen may be initially neuroprotective but may later become neurotoxic during the progress of PD [20]. Studies have shown that IL-12 levels are elevated in the brains of individuals with PD. Additionally, animal studies have shown that blocking IL-12 can protect against the loss of dopamine-producing neurons and improve motor function in models with PD. One study showed that microglia significantly enhanced the progression of 1-methyl-4-phenyl-1,2,3,6-tetrahydropyridine (MPTP)-induced dopaminergic neurodegeneration. The relationship between microglial activation and neurodegeneration demonstrated that the indirect activation of microglial activation through induced toxicity underlies microglia-enhanced neurotoxicity [21]. TGF-β signaling has a role in neuronal maintenance, function, degeneration, and other signaling pathways [22,23]. TGF-β1 is upregulated in CNS inflammation, neural apoptosis, synaptic deficits, and neurodegeneration for PD and AD [24]. TGF-β1 and 2 increased in PD patients compared to the control. This study has provided that TGF-β1, together with IL-6, is essential for de novo differentiation of IL-17-producing T cells from naïve CD4T cells in vitro [25,26]. It creates an environment that favors the development of Th17 cells while suppressing the differentiation of Th1 and Th2 cells. The presence of TGF-β1 is essential for the expression of the transcription factor RORγt, which is necessary for Th17 cell differentiation. When the naïve T cell is activated in the presence of TGF-β alone, it expresses Foxp3 and differentiates into a regulatory T cell, whereas, in the presence of TGF-β1 plus IL-6, the same cell expresses ROR-γt and differentiates into pathogenic Th17 [25,27].

*Mucuna pruriens* (L.) DC. (MP), a tropical plant commonly known as velvet bean, has been traditionally used in herbal medicine to treat PD [28]. Recent studies have investigated the neuroprotective effects of MP in treating PD, and it has been suggested that the presence of L-DOPA, a precursor to the neurotransmitter dopamine, is a key factor in its effectiveness. Another recent study showed an improvement in PD symptoms using Japanese *Mucuna pruriens* and proved its effectiveness in alleviating the symptoms of the disease without increasing dyskinesia [29].

In this study, we used standard L-DOPA as a positive control drug to validate the effectiveness of MP in improving the symptoms of PD. MP contains a high concentration of L-DOPA [28], with a ratio of 1:20 compared to standard L-DOPA medication used to treat PD [30].

We hypothesized that the oxidative stress and damage to dopaminergic neurons induced by rotenone, which eventually leads to neuroinflammation, motor and non-motor deficits in PD pathogenesis [17,31,32], and could be ameliorated equally by the MP and L-DOPA when these two are used in a comparative ratio of 1:20. The objective of our study was to evaluate the neuroprotective effect of MP and L-DOPA by investigating certain cytokines and to understand the underlying mechanism of how these work at the immunological level to reverse the symptoms of PD in mice. For that purpose, Il-6, TGF-β1, and IL-12p40 were chosen as cytokines of interest concerning the neuroprotective effects of MP and L-DOPA, which have not been investigated in the rotenone-intoxicated mouse model yet. If MP and L-DOPA can successfully alleviate the level of TGF-β1 and IL-6 in mice serum, it might reduce the PD symptoms by reducing the level of Th 17 in blood. The beam balance test and olfactory test were also used to investigate the progression and alleviation of PD symptoms. This study was performed as preliminary work to investigate the effect of MP as an anti-parkinsonian remedy in comparison to synthetic L-DOPA, which is clinically used to treat PD by specifically focusing on IL-6, IL-12p40, and TGF-β1.

## 2. Materials and Methods

### 2.1. Agents

Rotenone (Sigma-Aldrich, Seoul, Republic of Korea) was suspended in 1% DMSO (Sigma-Aldrich) and diluted with distilled water. In 2000, the Greenamyre group reported that systematic rotenone could reproduce the two pathological hallmarks of PD as well as certain parkinsonian motor deficits [33]. Keeping in mind these findings, rotenone was chosen as a drug of choice to induce the symptoms of PD in mice used in this experiment.

### 2.2. Animals and Ethical Approval

Twelve male C57BL/6 mice (6 weeks old; weighing 20–22 g) were used. Mice were purchased from Cronex (Nohyeong-ro, 307, Jeju, Republic of Korea). All animals were acclimatized for one week under standard conditions (room temperature 22 ± 2 °C and 12 h light/dark cycle). Water and food were allowed ad libitum. All the experimental protocols were officially approved by the institutional animal care and use committee at the Jeju National University (approval number 2023-0018).

### 2.3. Mucuna pruriens (*L.*) DC. and L-DOPA with Dose Verification

*Mucuna pruriens* (L.) DC. sample was obtained from the botany department of Quaid-e-Azam University, Pakistan. A simple water extract of MP was developed based on the presence of approximately 5% L-DOPA content in MP [30]. MP powder was first ground finely. The sample was then mixed in distilled water and centrifuged for 30 min at 4000 rotations per minute. The supernatant was extracted, filtered, and stored at 4 ℃. This stock solution was not used for longer than 14 days. Treatments were given through intraperitoneal injection. L-DOPA was used as a positive control drug in this experiment and set to 1:20 with respect to the MP drug. This ratio was chosen because the MP contains 5% L-DOPA by weight. Both L-DOPA and rotenone were administered intraperitoneally to the mice. A total of 25 mg/kg L-DOPA was used in relation to the 500 mg/kg MP dose.

### 2.4. Parameters to Validate PD Induction and Treatment

#### 2.4.1. Behavioral Assessment

The following tests were performed to evaluate motor dysfunction and non-motor dysfunction in PD-infected mice. All the mice underwent these tests with a gap of 30 min. Tests were performed on days −10, day −20, and day −28 of the Parkinson’s induction period and on day 7, day 13, and day 19 of the PD treatment period.

##### Beam Balance Test

To check the volume strength and coordination of the animal, a beam balance test was performed with a 60 cm bar. Animals were made to walk on a 6 mm diameter bar for analysis of motor coordination. The distance of the bar from the ground was almost 50 cm to avoid mice jumping off. To perform the beam balance test, the animal was held in front of the apparatus. The animal was slightly pushed backward for about 10 cm to align the body perpendicular to the bar and release the tail simultaneously while starting the clock. If the animal fell before 5 s had passed, the experiment was repeated.

##### Olfactory Test/Buried Pellet Test

Olfactory disturbances are one of the early non-motor symptoms observed in PD and show impairment in odor detection, differentiation, and identification [34]. To perform the olfactory test, a cookie (custard and chocolate cookie, no brand, Jeju si, Republic of Korea) was buried approximately 0.5 cm below the mouse cage. A clean mouse cage with 3 cm of clean bedding was used for this experiment. The mouse was placed in the center of the cage, and then the latency to dig up to eat the cookie was measured using a stopwatch. If the animal could not locate the cookie within 5 min, a score of 5 min was given. Bedding was changed for every mouse, and cookies were buried in different locations on each test day. The latency to locate the pellet was averaged after performing the experiment 3 to 5 times and then compared with the results of the healthy animal.

#### 2.4.2. Enzyme-Linked Immunosorbent Assay

Mouse IL-12, IL-6, and TGF-β1 enzyme-linked immunosorbent assay (ELISA) kits were purchased (Thermofisher and Sigma Aldrich, Jeju si, Republic of Korea). All the experimental procedures were performed following the instructions recommended by the manufacturer. The cytokine concentration was calculated using standard protein concentration.

### 2.5. Rotenone Model Induction and Treatment Plan

A total of 12 C57BL/6 mice (male) were randomly divided into the control group (n = 3) and the model group (n = 9). The model group mice were intraperitoneally administered rotenone solution (30 mg/kg) [31] every day for a period of 28 days [35]. At the same time, the control group received an intraperitoneal administration of 1% DMSO solution. The blood of the mice was taken on the 28th day. Nine mice in the model group were again divided into 3 groups randomly 28 days before the start of the treatment period in the following manner: the PD-infected control group (n = 3); the PD-infected group treated with MP (n = 3); and the PD-infected group treated with L-DOPA (n = 3).

### 2.6. PD Induction Score

PD induction score was formulated to validate the induction of PD in mice. The criteria chosen to make scores were the balance on the beam, hind-limb skipping, olfactory sensation, and weight loss. The mice showing normal behavior in the above-mentioned conditions were given a score of 0, while mice with mild, moderate, and severe anomalies in those conditions were given scores of 1, 2, and 3, respectively. After gathering all the data, the scores were summed up for every mouse to validate PD induction. These data were further used to plot the DP induction score on the chart.

### 2.7. Experimental Design

**Figure 1:**
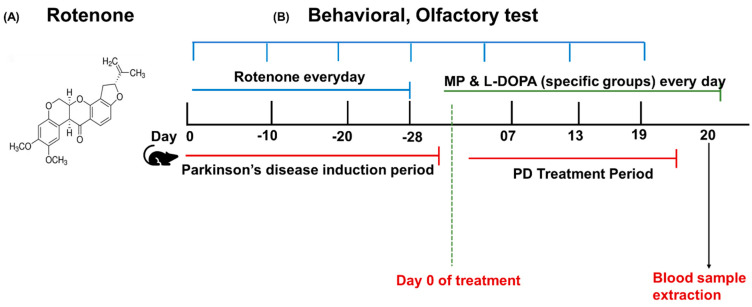
(**A**) Chemical structure of rotenone, (**B**) Experimental protocol of the whole procedure. Behavioral tests were performed on days 10, 20, and 28 of Parkinson’s disease induction period and on 7th, 13th, and 19th days of Parkinson’s disease treatment period. Blood samples were taken on day 28 of PD induction period and then on day 20 of PD treatment period to detect the level of IL-6, IL-12p40, and TGF-β1 in samples. Animals were sacrificed on 20th day after taking blood samples.

The whole experimental design of this study is shown in Figure 1B. Mice were administered rotenone for 28 days to induce the PD model. Then, these mice were randomly divided into 3 groups for treatment. In the first 28 days, the PD model group showed a significant difference in motor and olfactory functions than the control group. After 28 days, PD mice were given MP and L-DOPA treatment for a period of 19 days. The chemical structure of PD-inducing drug rotenone is shown in Figure 1A.

### 2.8. Statistical Analysis

The statistical analysis was performed using the OriginLab 2018 version. A two-way paired *t*-test was performed on the data obtained in this study. Results are expressed as the mean ± SD. *p*-value < 0.05 was considered statistically significant.

## 3. Results

### 3.1. Rotenone-Induced Parkinson’s Disease (PD) Symptoms in Mice

**Figure 2:**
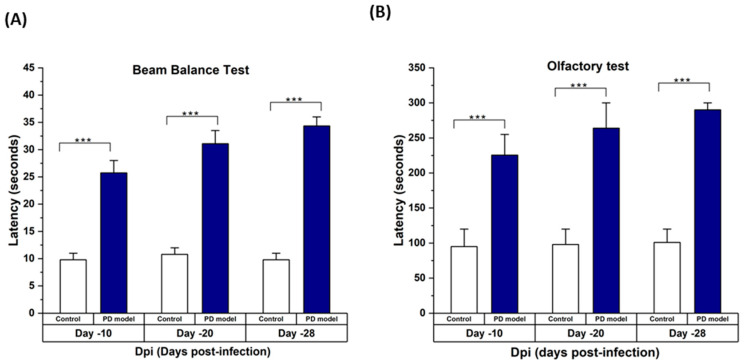
(**A**) Rotenone-intoxicated C57BL/6 mice showed reduced motor strength based on the beam balance test. Control mice were intraperitoneally injected with 1% DMSO, and PD mice were injected with rotenone 30 mg/kg daily for 28 days to induce Parkinson’s symptoms. The beam balance test was performed on days −10, −20, and −28 to assess the degree of PD symptoms. (**B**) Rotenone-intoxicated C57BL/6 male mice showed reduced motor strength based on the beam balance test. Control mice were intraperitoneally injected with 1% DMSO, and PD mice were injected with rotenone 30 mg/kg daily for 28 days to induce Parkinson’s symptoms. The beam balance test was performed on days −10, −20, and −28 to assess the degree of PD symptoms. Statistical comparison by two-way paired *t*-test between the control vs. PD model was performed. Data represent the means ± SD. *** *p* < 0.001.

**Figure 3:**
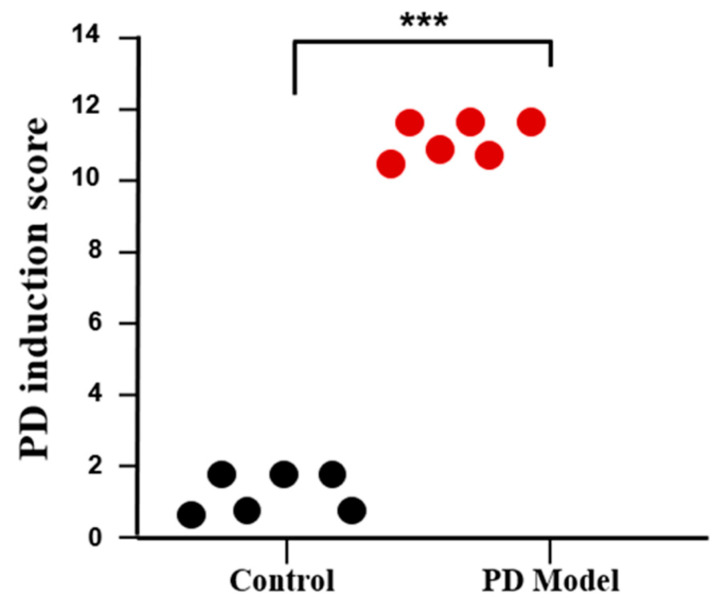
PD induction score in mice. The parameters used to design the PD induction score were balance test on the beam, hind-limb skipping, weight loss, and olfactory sensation. 0 score was given when no symptoms were present. Scores of 1, 2, and 3 were given when mild, moderate, and severe symptoms were present, respectively, for each of 4 tests and added together for PD induction score. Data represent the means ± SD. *** *p* < 0.001.

The mice intoxicated with rotenone underwent different behavioral tests. Mice showed a significant difference in latencies of the behavioral tests. It was found that rotenone caused a marked decrease in motor coordination in the mice. It was also observed that rotenone-intoxicated mice showed higher latency in finding the hidden cookie. Figure 2A,B shows the results of the beam balance test and olfactory test, respectively. These tests performed on different days confirmed the progression of symptoms of PD in mice. Figure 3 shows the PD induction score, which confirmed the significant difference in scores of healthy and PD mice.

### 3.2. Comparative Study of MP and L-DOPA in Improving the Symptoms of Parkinson’s Disease

#### 3.2.1. Effects of MP and Improving the Physical Symptoms of PD in Comparison to L-DOPA

**Figure 4:**
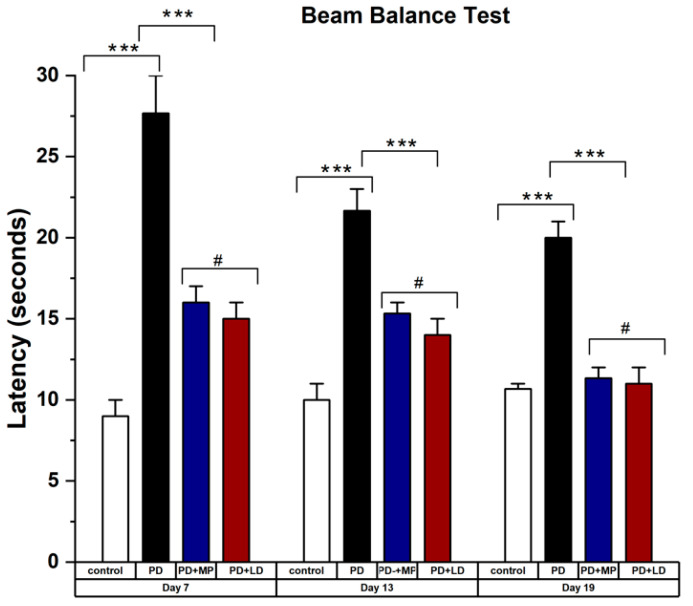
Rotenone-intoxicated mice, when treated with MP and L-DOPA, reduced motor dysfunction in PD mice measured through beam balance test. Total crossing time was significantly reduced in PD mice treated with MP and L-DOPA. Statistical comparison by two-way paired *t*-test between control vs. PD, PD vs. MP, and MP vs. L-DOPA. Data represent the means ± SD. *** *p* < 0.001, # Non-significant *p*-value. n = 3 mice per group. Beam balance test showed relative improvement in latency even in the PD group not treated by MP or L-DOPA, which is due to the body’s self-defense mechanism, which tries to get rid of the toxins.

**Figure 5:**
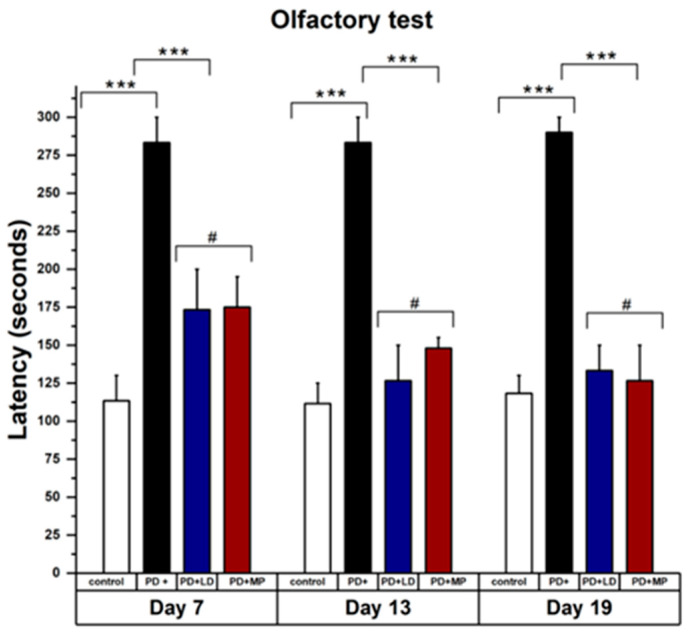
MP and L-DOPA treatments of the PD mice improved olfactory impairment. PD mice treated with MP and L-DOPA showed significant improvement on olfactory test on day 7, but the improvement shown on day 13 and day 19 was not much different from the initial results. On the other hand, there was no improvement in the latency shown by PD mice, which were not treated with either MP or L_DOPA, which is very different from beam balance test. Statistical comparison by two-way paired *t*-test between control vs. PD, PD vs. MP, PD vs. L-DOPA, and MP vs. L-DOPA. Data represent the means ± SD. *** *p* < 0.001; # Non-significant *p*-value. n = 3 mice per group.

The effects of MP and L-DOPA showed non-significant differences in the data analysis. Figure 4 shows the results of the beam balance test taken on days 7, 13, and 19, with significant improvement in PD mice after being treated with MP and L-DOPA. The values from MP and L-DOPA were not significantly different. Figure 4 also shows improvement in beam balance tests performed on days 13 and 19, even in non-treated PD mice. Figure 5 shows the results of the olfactory test showing significant improvement in PD mice treated with MP and L-DOPA. Latency for olfactory impairment stayed similar, 280 s, from day 7 to 19 for PD induced by L-DOPA, while latency from balance test decreased from 28 s to 20 s. It is noted that olfactory symptoms started early in PD mice and were not easily restored naturally.

#### 3.2.2. Effects of MP in Improving the Inflammation of PD in Comparison to L-DOPA

**Figure 6:**
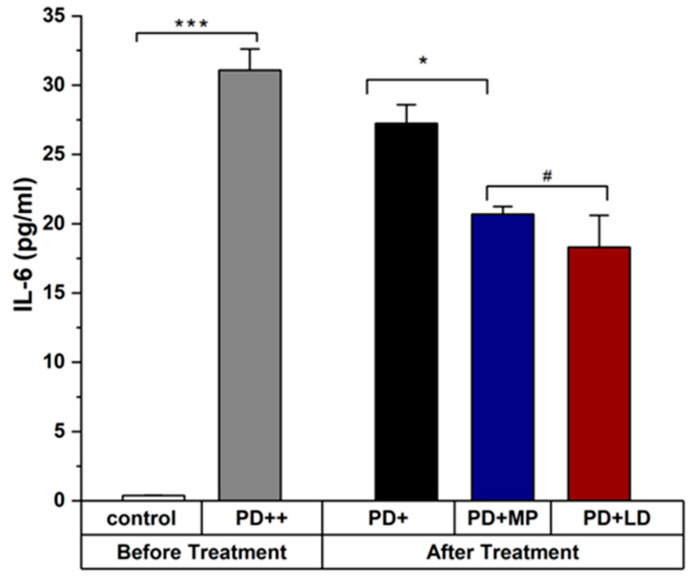
Level of IL-6 in control, Parkinson, and treatment groups. There was a significant difference between the level of IL-6 in control and rotenone-intoxicated mice (PD). IL-6 in MP and L-DOPA-treated mice showed non-significant differences in their values. The levels of IL-6 shown in “Before Treatment” section of the graph were measured using blood sample taken on day 28 of PD induction, while the level of IL_6 shown in “After Treatment” section of the graph was measured using blood samples taken on day 19 of PD treatment. Data represent the means ± SD. *** *p* < 0.001; * *p* < 0.05; # Non-significant *p*-value. n = 3 mice per group. IL-6 level after treatment shows its level in mice serum at day 19 of the treatment period in untreated and treated PD groups.

**Figure 7:**
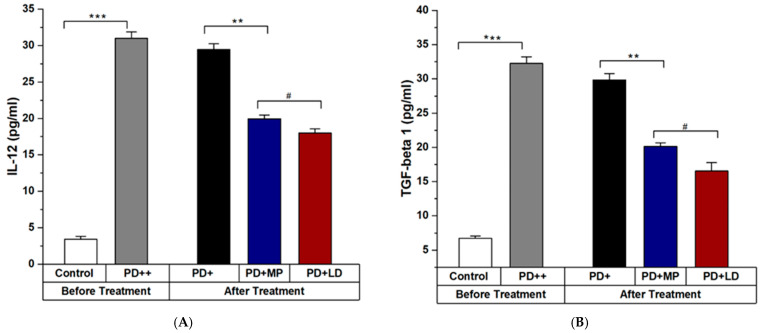
(**A**) Level of IL-12 in control, Parkinson, and treatment groups. (**B**) Level of TGF-β1 in control, Parkinson, and treatment groups. There was a significant difference between the level of IL-12 and TGF-beta 1 in control and rotenone-intoxicated mice (PD). IL-6 in MP and L-DOPA-treated mice showed non-significant differences in their values. The levels of IL-12 and TGF-β1 shown in “Before Treatment” section of the graph was measured using blood sample taken on day 28 of PD induction. While the level of IL_12 and TGF-β1 shown in “After Treatment” section of the graph was measured using blood samples taken on day 19 of PD treatment. Data represent the means ± SD. *** *p* < 0.001; ** *p* < 0.01; *; # Non-significant *p*-value. n = 3 mice per group. IL-12 and TGF-β1 levels after treatment show their levels in mice serum at day 19 of the treatment period in untreated and treated PD groups.

The expression levels of TGF-β1, IL-6, and IL-12 from ELISA analysis at day 19 of treatment show significant alleviation in Figure 6 and Figure 7. The serum analysis of mice confirmed that the PD mice were treated with MP and L-DOPA. L-DOPA and MP have similar results in improving the symptoms of PD when used in a ratio of 1:20. Please describe the results quantitatively using statistical analysis. Figure 7 should include (A) and (B).

The small standard deviation (SD) values observed in our experiments suggest consistent and precise measurements across the samples. However, given the small sample size, these results should be interpreted with caution, and further studies with larger cohorts are needed to validate these findings.

## 4. Discussion

In this study, we investigated the neuroprotective effect of MP and L-DOPA on the rotenone-intoxicated C57BL/6 mice. For that purpose, we administered rotenone intraperitoneally at a dose of 30 mg/kg body weight daily for 28 days to induce PD symptoms. Rotenone can cross the blood–brain barrier and inhibit the activity of complex-1 in the mitochondrial respiratory chain. It also increases the oxidative stress and neuroinflammation while decreasing the level of neurotransmitters. In our study, rotenone intoxication significantly decreased motor coordination, olfactory impairment, and increased neuroinflammation, which was confirmed by the beam balance test, olfactory test, and ELISA analysis, respectively. These results were in corroboration with other studies, which also observed impairment in behavioral and inflammatory parameters [36] and confirmed the induction of PD in mice. L-DOPA and MP were used to treat PD during the PD treatment period. PD-treated mice showed significant improvement in their symptoms and reduction in the level of pro- and anti-inflammatory cytokines when compared with non-treated PD mice. Furthermore, both L-DOPA and MP-treated mice showed similar improvements with no significant differences (*p* < 0.05) when used in a 1:20 ratio.

During PD induction, serum levels of IL-6, TGF-β1, and IL-12 were significantly high, which confirmed the induction of the PD through rotenone [11,12,16,25]. IL-6 is a crucial biomarker of PD progression and PD improvement. Furthermore, TGF-β1 and IL-12 are important biomarkers of neuroinflammation. The olfactory impairment also appeared in mice, validating the previous studies that olfactory impairment is one of the early symptoms of PD [37]. It is important to note that PD mice, even without any treatment, also showed improvement in all tests performed, except olfactory latency. The reason behind this improvement may be the body’s self-defense mechanism of the mice, which tries to get rid of the toxins. It is important to consider this factor when evaluating the efficacy of treatments such as MP and L-DOPA. Future studies will extend the observation period to better differentiate between treatment-induced improvements and spontaneous recovery. Observing traits such as motor function, cognitive abilities, and neuroinflammation levels beyond the initial treatment period will provide a more comprehensive understanding of the therapeutic potential and sustainability of MP treatment in PD models.

Neuroinflammation plays a critical role in the progression of PD. The outcome of our study showed that the administration of rotenone resulted in a significant elevation in the levels of IL-6, TGF-β1, and IL-12. These cytokines can directly activate inflammatory and apoptotic pathways, causing neuroinflammation. The observation in our current study is in agreement with previous studies, showing increased levels of these cytokines in the neuroinflammatory process [11,25,38]. Treatment with MP (500 mg/kg/day IP) and L-DOPA significantly reduced the level of specific cytokines in the serum of the mice. The valid dose of MP for the experiment was determined by comparing it with the standard L-DOPA drug, using a 1:20 ratio to account for the presence of available L-DOPA in both compounds. Because the active ingredient in MP responsible for these effects is L-DOPA, which is a precursor to dopamine, a neurotransmitter that is deficient in PD. By supplementing with L-DOPA, MP can help to restore dopamine levels in the brain and alleviate the symptoms of PD.

The reason for choosing the IL-6 along with TGF-β1 is to further investigate the effect of L-DOPA and MP on ameliorating the level of pathogenic Th 17 in the blood. If the levels of IL-6 and TGF-β1 are collectively increased in the blood, they can also increase the level of pathogenic Th 17 in the blood by changing CD4 cells into pathogenic Th 17.

Furthermore, it is widely accepted that mice Th17 cells originate from CD4+ T cells in the presence of IL-6 and TGF-β1, and their development is then stabilized and/or amplified by IL-23. In this study, we selected IL-12p40 for our serum analysis due to its shared presence in both IL-12 and IL-23 cytokines. IL-12 is composed of p40 and p35 subunits, while IL-23 is composed of p40 and p19 subunits [39]. Given that IL-23, a member of the IL-12 cytokine family, plays a critical role in the differentiation of Th17 cells [39], the use of IL-12p40 allows us to simultaneously assess the involvement of both IL-12 and IL-23 in the pathogenesis of the disease process under investigation. This dual functionality of the p40 subunit makes it a key marker, providing a broader understanding of the cytokine’s role in immune responses, which can be further validated in future studies. In this study, L-DOPA and MP downregulated the level of IL-6 in PD mice serum; so, it can be concluded with scientific evidence that L-DOPA and MP can effectively reduce the level of pathogenic Th 17 in the serum of animal models intoxicated with PD-induced chemicals. However, these findings need a long-term analysis of the serum and flow cytometry to validate the results. This can be an interesting area of research, especially in the field of phytochemicals and how MP can work at the immunopathological level to ameliorate the symptoms of neurodegenerative diseases.

### Future Work and Limitations

In this study, the small sample size in each group and the relatively brief treatment duration were acknowledged as limitations. These choices were made to conduct an initial investigation into the potential of MP in treating Parkinson’s disease (PD). While the preliminary findings are promising, further studies with larger sample sizes and extended treatment periods are essential to validate these results and ensure their robustness and reproducibility. Additionally, the shorter treatment duration may not fully capture the long-term effects of MP and L-DOPA, which could be more apparent in extended studies.

Another key aspect not covered in this study is the aggregation of alpha-synuclein in the brain, a critical marker for PD pathology. Future research should include immunofluorescence analysis of brain samples to assess alpha-synuclein accumulation, providing a more comprehensive understanding of PD induction and the neuroprotective effects of MP and L-DOPA. Incorporating these elements in follow-up studies will help to confirm and extend the findings reported here.

## 5. Conclusions

We investigated the comparative analysis of the anti-parkinsonian role of MP and L-DOPA in PD. We developed an in vivo model of PD in C57BL/6 mice using rotenone and examined the therapeutic effects of MP and L-DOPA in ameliorating the disease symptoms by reducing neuroinflammatory cytokines. The parameters for validation of PD induction and evaluation of the treatments included motor and olfactory impairment at a physical level, as well as serum biomarker analysis at pathological examinations. For the serum biomarker analysis, we selected pro-inflammatory and anti-inflammatory cytokines, namely, IL-6, TGF-β1, and IL-12p40. MP was chosen as the treatment drug due to its proven advantages against PD, primarily due to the presence of L-DOPA. To fully understand its effectiveness, L-DOPA was selected as a positive control drug to compare the results. Our findings report that MP can attenuate motor and olfactory dysfunction in rotenone-intoxicated PD mouse model and downregulate IL-6, IL-12p40, and TGF-β1 in mice serum, suggesting its effective role in anti-parkinsonism. These findings highlight the capability of MP to improve PD symptoms by compensating for the loss of dopamine and activating the dopaminergic neurons. In the future, we aim to further explore the neuroprotective effects of MP using other neuroinflammatory biomarkers such as TNF-alpha alpha-synuclein. Also, we aim to explore the scope of this study on how MP, along with low-intensity therapeutic ultrasound, can alleviate the symptoms of PD in rotenone-intoxicated mice.

## Data Availability

Data are available upon request to the corresponding author.
